# Diversity of Glutathione S-Transferases (GSTs) in Cyanobacteria with Reference to Their Structures, Substrate Recognition and Catalytic Functions

**DOI:** 10.3390/microorganisms8050712

**Published:** 2020-05-11

**Authors:** Mohandass ShylajaNaciyar, Lakshmanan Karthick, Peter Arul Prakasam, Garlapati Deviram, Lakshmanan Uma, Dharmar Prabaharan, Sushanta Kumar Saha

**Affiliations:** 1 Department of Marine Biotechnology, School of Marine Sciences, National Facility for Marine Cyanobacteria (Sponsored by DBT, Govt. of India), Bharathidasan University, Tiruchirappalli 620 024, Tamil Nadu, India; shylajanachiyar@gmail.com (M.S.N.); karthick.lakshman@gmail.com (L.K.); bioinarul@gmail.com (P.A.P.); deviramgarlapati@gmail.com (G.D.); uma@bdu.ac.in (L.U.); 2 Shannon Applied Biotechnology Centre, Limerick Institute of Technology, Moylish Park, Limerick V94 E8YF, Ireland (ROI); Sushanta.Saha@lit.ie

**Keywords:** cyanobacteria, glutathione S-Transferases (GSTs), detoxification

## Abstract

Glutathione S-Transferases (GSTs) comprise a diverse group of protein superfamily involved in cellular detoxification of various harmful xenobiotics and endobiotics. Cyanobacteria, being the primordial photosynthetic prokaryotes, served as an origin for the evolution of GSTs with diversity in their structures, substrate recognition, and catalytic functions. This study analysed the diversity of GSTs in cyanobacteria for the first time. Based on the sequence alignment and phylogenetic tree analysis, 12 GST classes were identified, which are distributed variedly within cyanobacterial orders such as four in *Pleurocapsales*, eight in *Chroococcales*, seven in *Oscillatoriales*, five in *Stigonematales*, and nine in *Nostocales*. Detailed evolutionary analysis of cyanobacterial GSTs suggested that the order *Pleurocapsales* served as the ancestry for GST evolution. The analysis also identified a conserved motif S[GLNTARS][ADE]I[LAI] with signature residues, cysteine, serine, and tyrosine at the N-terminal end that serves as the initiating residue for detoxification. Alternatively, the grouping of cyanobacterial GSTs and their unique signature residues were located, which serve as a possible discriminating factor. The study also described the mode of glutathione binding between the identified cyanobacterial GST groups highlighting the differences among the GST classes. New GST sequence data may improve further our understanding on GST evolution and other possible divergences in cyanobacteria.

## 1. Introduction

Glutathione (GSH, reduced form) metabolism is considered as ancient as the history of life, dating back to the evolution of the oxygen-containing atmosphere [1]. Several defence mechanisms have evolved to remove the oxidizing agents that allow the organisms to adapt and survive, of which the enzyme Glutathione S-Transferase (GST)-mediated reaction plays a pivotal role in cellular detoxification of harmful xenobiotics and endobiotics [2]. GSH and GSTs are believed to have evolved in response to the increase in oxygen in order to scavenge the generated reactive oxygen species [3].

Generally, detoxification enzymes act as a first line of defence against the environment characterized by the presence of toxins and pollutants. The detoxification process is performed in three phases, phase I enzyme oxidation, reduction or hydrolysis of the substrate, introducing a reactive group that can be attacked by phase II enzymes, which conjugate the active substrate with small molecules making the conjugate more water-soluble for excretion from cells by phase III enzymes [4]. Among these key enzymes, GSTs represent an integral part of the phase II step and detoxify xenobiotics through catalysing the nucleophilic attack by GSH on electrophilic carbon, sulphur, or nitrogen atoms of nonpolar xenobiotic substrates, thereby preventing their interaction with crucial cellular proteins and nucleic acids. This generates water-soluble glutathione conjugates linked by a thioether bond (GS-R) that can then be degraded, or excreted out of the cell [5,6].

GSTs are the largest superfamily of proteins found almost in all eukaryotes and restricted to certain prokaryotic bacteria including cyanobacteria, proteobacteria, phototrophs and a few Gram-positive bacteria [7]. GSTs are divided into three different families: (i) cytosolic GSTs, (ii) mitochondrial GSTs, and (iii) microsomal (membranous) GSTs designated as MAPEGs (a membrane-associated protein involved in ecosanoid and glutathione metabolism) [4]. Cytosolic GSTs, which constitute the largest GST family are currently recognized as alpha, beta, delta, epsilon, zeta, theta, lambda, mu, nu, pi, sigma, tau, phi, and omega [4,8]. Among cytosolic GST families, members of the same class possess sequence identity greater than 40%, whereas 20% identity was found between classes [1]. The mitochondrial GSTs are an evolutionary distant form of cytosolic GSTs that constitute different topology and subcellular localization [9]. Distant classes of GSTs identified in bacteria are nu, zeta, and eta [10,11] and in cyanobacteria are chi [12], and rho [13].

The structural features of GSTs are composed of two ligand binding sites, the first G-site specific for GSH, which possess a conserved group of amino acid residues in the amino-terminal domain and the second is the substrate binding H-site in the carboxyl-terminal domain, which is structurally much more variable among GST classes [4]. The functional properties of amino acid residues forming the G-site are highly conserved among the different classes of GSTs, while the binding pocket of the H-site varies considerably among GST classes. This diversity governs the electrophile substrate specificity, ability to pick, and catalyse a large number of structurally variable substrates [14].

Cyanobacteria are unique prokaryotes, which exhibit oxygenic photosynthesis and produce reactive oxygen species (ROS) as a result of photosynthesis. GSH and the GSH-dependent GST enzymes evolved concurrently to protect themselves against the ROS which they massively produce by their active photosynthesis. These cyanobacteria were reported to possess high concentrations of GSH in their cytosol and might have been the very first organism to harbour GSH-utilizing enzyme GST [15]. Cyanobacteria are highly diverse with a comprehensive set of different morphotypes from primordial unicellular form to multicellular heterotrichous form with genomic DNA ranging from ~1.2 to ~12 MB [16]. These large diversities may have a direct impact on the distribution and evolution of cyanobacterial proteins including GSTs. However, cyanobacterial GSTs have been poorly studied.

Though, cyanobacteria may be very well the first organism to have harboured GSH utilizing enzyme, ironically its GST has not been studied extensively so far. The puzzle of the evolution of the cyanobacterial GSTs and the lines between the groups to date is incomplete. Considering this fact, an attempt was made in this study, through genome-wide analysis and by examining the multiple characteristics such as distribution, motif or signature patterns available in the sequences using the phylogenetic approach. Nevertheless, details about the GST super-families in cyanobacteria are essential to gain deeper insight information about the GSTs versatility in detoxifying the diverse compounds. For this purpose, an extensive study was carried out to seek the complex pattern, divergence, and distribution of GSTs in the cyanobacterial orders.

## 2. Materials and Methods

### 2.1. Sequences Retrieval

A dataset contain genome information of 126 cyanobacteria used by Shih et al. [17] was selected for analysis in this study. The 126 cyanobacteria were comprised of *Pleurocapsales* (21), *Chroococcales* (52), *Oscillatoriales* (29), *Nostocales* (18), and *Stigonematales* (6). Then, 405 GST protein sequences from 126 cyanobacteria were manually retrieved from the NCBI complete genome database and the Cyanobacterial Knowledgebase [18] using the search term Glutathione S-Transferase/GST. Further repeated and truncated sequences hailing from the same organism were removed manually. Generally, cyanobacteria were grouped based on morphological complexity and taxonomic studies into morphologically distinct evolutionary forms. For better understanding and accuracy, the alignment and analysis of the GST sequences were separated into five orders: order I-*Chroococcales*, order II-*Pleurocapsales*, order III-*Oscillatoriales*, order IV-*Nostocales*, and order V-*Stigonematales* [17].

### 2.2. Sequence Alignment and Phylogenetic Analysis

The separated sequences were aligned globally using two different tools: MAFFT [19] and CLC Genomic workbench 8.5.1 (CLC Bio-Qiagen, Aaarhus, Denmark). The sequences which hold a lot of discrepancies in the alignment were removed manually (83 out of 405). To improve the phylogenetic inferences, the poorly aligned regions were tuned using trimAL [20].

A phylogenetic tree was constructed for each order separately for analysis and for all the best available 322 GST sequences. The tree was constructed using MEGA 5 [21]. Phylogenetic reconstruction was performed using the maximum likelihood statistical method with 500 replicates bootstrap. The substitution model used was Jones–Taylor–Thornton method using the rates and pattern as Gamma Distributed (G). The constructed trees were visualized and analysed using FigTree v1.3.1 [22]. As GSTs belong to multigeneic families, out-group identification is unsystematic hence phylogenetically related outgroups for each family were not described. Hence, no out-group was included in the present analysis. Evolutionary relationships among the cyanobacteria orders were inferred from the amino acid based Neighbor-Joining phylogeny that was constructed by Jones– Taylor–Thornton method using the rates and pattern as Gamma Distributed (G) using MEGA 5.0.

### 2.3. Identification of Conserved Features

“Serine” of SNAIL/TRAIL motif at the N-terminal end, reported to provide a polar functional group for GSH binding, is highly conserved in all GST sequences, which also serves as a signature for marking the sequence as GST [23]. Hence, by using SNAIL/TRAIL as reference motif, the hunt for similar architecture in 322 retrieved cyanobacterial GST was carried out manually within the aligned sequences. Sequence alignment was carried out as mentioned in Section 2.2. A common unique GST motif architecture that comprises 12 motifs was identified, and those sequences were subjected to further analysis. The presence of reference motif in the sequence was marked as GST and the sequences lacking the reference motif were removed manually. GST sequences belonging to each motif were retrieved manually and assembled for each class which were further cross verified with phylogenetic clade analysis. Further, the presence of sequence-specific signature or conserved residues namely cysteine, serine, and tyrosine were evaluated for their presence. This analysis was carried out by grouping the sequence into Y, S, and C type GSTs. Founded on this analysis and extensive visual inspection, all alignments were analysed for group-specific signature sequences.

### 2.4. Construction of Network

A sequence similarity network for the GST sequences was performed using the Enzymes Function Initiative-Enzyme Similarity Tool (EFI-EST) [24] and the resulting network was visualized using Cystoscope 3.4.0 using the organic layout [25]. The 322 cyanobacterial GST sequences were those clustered within a sequence similarity network at a BLAST E-Value of 1 × 10^−11^. The thresholds used in the static network in this study were chosen to illustrate best the major subgroups and classes from each other.

### 2.5. GST Structure Prediction and Molecular Docking

cyGSTX1, cyGSTX6, and cyGSTX7 belonging to Y (tyrosine), S (serine), and C (cysteine) types were chosen as representatives for comparative modeling analysis. Models were built with MODELLER 9.10 (http://salilab.org/modeller/) and the best models were selected based on the DOPE scores. The model structure thus obtained was subjected to refinement via energy minimization using VMD1.9.2. CHARMM27 force field and Nanoscale Molecular Dynamics (NAMD) was used and minimization was performed with 100000 steps steepest decent with scaling 0.6 and cut off 8.0. The best frame thus obtained was refined by NAMD minimization for 5000 iterations. After minimization, the root mean square deviation (RMSD) of the backbone atoms was calculated with reference to the starting structure. The lowest energy structure was taken and this structure was subjected to interaction studies.

Macromolecular docking between GST and GSH was performed through Autodock4.2 [26]. Initially the grid box was set in accordance with the corresponding residues and saved as a grid parameter file (gpf). The docking simulation was performed using Lamarckian genetic algorithm (LGA) with a population size of 150, energy evaluation of 2,500,000, and search runs of 50 [27]. The structure with the lowest free energy of binding in a highly populated cluster was chosen as the optimal docking and subjected to interaction studies. The docking poses were visualized and analysed using the MOLEGRO Molecular Viewer (https://molegro-molecular-viewer.software.informer.com/2.5/) and the graphical representation was done with Chimera 1.6.2 (https://www.cgl.ucsf.edu/chimera/olddownload.html).

## 3. Results and Discussion

Many prokaryotic and eukaryotic GST proteins are classified into a collection of family-like classes mainly based on the sequence, structural similarities, and differences in the organization and composition of their active sites [28]. However, their distribution in each organism remains controversial. Cyanobacteria, a group of primordial prokaryotes have vast diversity in genome organization that serves as a major reason for the divergence of GST protein among the orders and make them promising targets for evolutionary analyses. GST sequences that possess less than 25% sequence similarity are classified as a different type and GSTs that share more than 40% sequence similarity are considered as the same class [4,29]. Considering the present scenario, we analysed the global distribution and phylogenetic relationship of GSTs among the cyanobacterial orders. Primary grouping of GSTs was performed based on the protein sequences and their degree of identity. The major criteria that support the classification and discrimination of GSTs among the cyanobacterial orders are as follows:

I. Presence of catalytically essential residues such as serine, tyrosine, and cysteine at the N- terminus [28]. The three catalytic residues present in the N-terminus of GST are broadly found in all types along with the GST specific motifs that contribute a polar functional group to the glutathione (G) binding site [30]. Changes in these signature residues result in functional variation, such as differences in catalytic properties, selective dimer formation, and substrate bindings.
II. Presence of the hydrophobic “lock and key” motif, the SNAIL/TRAIL motifs at the N-terminal end and presence of catalytic signature residue in C-terminal domain [23].
III. Difference in the organization and composition of the active site [31].

### 3.1. Cyanobacteria Contain Many Diverse Members of GSTs

**Figure 1:**
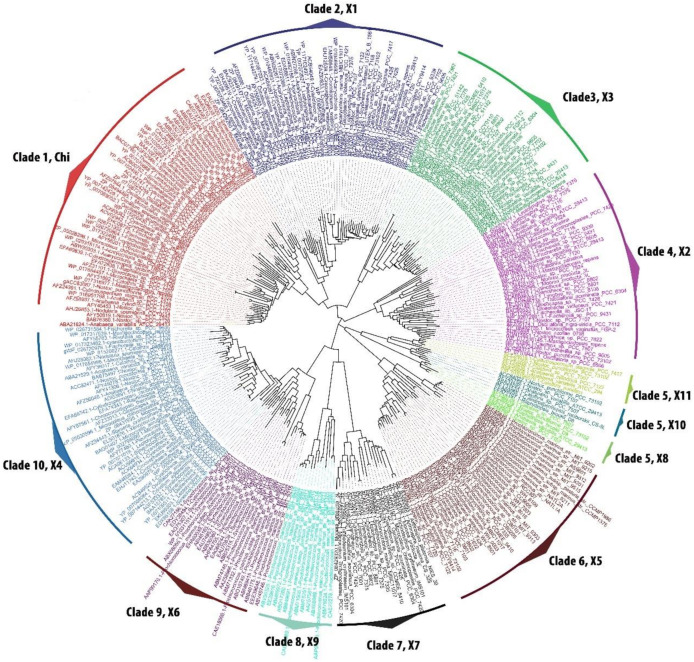
Phylogenetic tree of cyanobacterial glutathione S-transferase (GST) sequences belonging to the five orders. Cyanobacterial GSTs were clustered in 10 clades consisting of 12 GST classes. Mega5 software were used to construct the tree using maximum likelihood statistical method with 500 replicates bootstrap. The substitution model used was Jones–Taylor–Thornton method using the rates and pattern as Gamma Distributed (G). The constructed trees were visualized and analysed using FigTree v1.3. Each clade was analysed manually, named and colour codes were given to separate each clade which represents a type of GST. Chi, cyGSTChi; X1, cyGSTX1; X2, cyGSTX2; X3, cyGSTX3; X4, cyGSTX4; X5, cyGSTX5; X6, cyGSTX6; X7, cyGSTX7; X8, cyGSTX8; X9, cyGSTX9; X10, cyGSTX10; and X11, cyGSTX11.

Phylogenetic analysis of 322 GST sequences retrieved from 126 cyanobacteria [17] showed a total of 10 GST clades (one clade with ‘chi’ GST class and the remaining nine clades with 11 new GST classes) The most primordial unicellular form of cyanobacterial order *Pleurocapsales* was found in four GST clades, lower unicellular order *Chroococcales* presence was broadly observed in eight distinctly separated clades, middle filamentous order *Oscillatoriales* was found in seven conserved clades. Interestingly, higher heterocyst containing orders *Nostocales* and *Stigonematales* were found respectively in nine and five different clades (Figure 1). The distribution of common and order specific GST show the hierarchy of GST evolution among the cyanobacterial orders.

Since this was the first comprehensive data on cyanobacterial GST, for a clear understanding a naming convention was given to cyanobacterial GST for each class with reference to the previous names of prevailing GST. The nomenclature of cyanobacterial GST was framed using the lower case “cy” for cyanobacteria preceding the GST followed by the upper case denoting as “X”, then Arabic numeral denoting sub-types (1, 2, 3...) (cyGSTX1-cyGSTX11) (Table 1). The identified types which shared close similarities with already existing groups, were identified as “Chi” class (colour code-red). GSTs which possess partial similarity with other prokaryotic GSTs and specific forms standing as independent lineages were denoted as (cyGSTX1-cyGSTX11) (different colour code given to each type) (Table 1). Overall, the order *Nostocales* was the largest with nine different GST classes, followed by orders *Chroococcales, Oscillatoriales, Stigonematales,* and *Pleurocapsales* respectively with 8, 7, 5, and 4 classes (Table 1). This phylogenetic analysis of cyanobacterial GSTs suggests a gene duplication event, which led to the independent evolution of different GSTs type among the cyanobacterial orders.

### 3.2. Cyanobacteria Type Discrimination Based on Conserved Features

Putative 322 GST sequences retrieved from 126 cyanobacteria were subjected to alignment which showed the conservation of 12 motifs (M) namely M1-SGAIL, M2-SGAIL (2), M3-SLAIL, M4-SNAIL, M5-STEIA, M6-SDDII, M7-SAEII, M8-SSAIA, M9-SAIIN, M10-SLEII, M11-SAVIN, M12-SKDIL with the architecture of “ES [GLNTARS][ADE]I[LAI]” (Table 2) (Supplementary Figures S1,S2). The serine present in the identified motifs was found to be conserved in all identified GSTs. However, other amino acids in the motif, not directly involved in GSH binding, were highly diverse within the order (Table 2). This architecture was equivalent to SNAIL/TRAIL motif that is present in most GST classes that contribute polar functional groups to the GSH binding site [28].

Similarly, a putative SNAIL/TRAIL-like motif (SGAIV) at amino acid positions 86–90 was reported in the sequence of *Atu*GSTH1-1. The hydroxyl group of serine in motif SGAIV at position 86 forms a hydrogen bond with the γ-Glu portion of GSH, whereas the other residues of the motif are not directly involved in GSH binding [23]. Likewise, in cyanobacterial GSTs, serine in the motif architecture was found to be highly conserved in all the GST classes, but an amino acid change was observed within the motifs which is shown in Table 2. The N-terminal motif is an excellent target for discriminating the GST groups, because it is better conserved than others which possess the important part of the active site. However, it can further be powered by combining the presence of specific conserved motifs at the C-terminal end. The signature motif, found at the C-terminal end used for discriminating the cyanobacteria GST, is more degenerative.

Chi class reported as cyanobacteria specific is found in all orders possessing a unique motif SGAIL. Both Chi and cyGSTX1 GST groups share the same motif SGAIL (Table 2) and hence, the differentiation between Chi and cyGSTX1 is further refined by the presence of specific signature sequences at the N- and C-terminal ends. The two signature motifs in Chi group are GG[PA][KR]SRAS and NPFGK[VL]P[VA]L that are replaced by ISPN[SGN]RIP and BADIA[TC]YP in cyGSTX1. These motifs serve as an important feature for the discrimination of cyanobacterial GSTs of Chi from cyGSTX1 type. S(FL)AI(LM) and SNA(IVM)(LM) motifs are found in cyGSTX2 and cyGSTX3 groups, where the signature residue L is replaced by N in the cyGSTX3 motif. However, cyGSTX4 has the entirely unique motif ST(EDA)IA (Table 2). Likewise, cyGSTX5 possess the SD(DRV)I(IL) motif and cyGSTX6, cyGSTX7, and cyGSTX9 found in *Chroococcales, Oscillatoriales*, and *Pleurocapsales* possess SA(DE)II, S(ST)AI(AC), and SL(ED)I(IM) motifs respectively (Table 2). The similarities in the motifs indicate the close functional relatedness and the ladder of the GST evolution among the cyanobacterial orders. *Nostocales*, one of the evolved forms of cyanobacteria has been proved to have greater evolutionary divergence [32]. Three order specific cyGSTX8, cyGSTX10, and cyGSTX11 found in the higher order *Nostocales* have SAI(IV)N, SA(IV)IN, and SKDIL motifs which remain entirely specific from the other GST group’s motif. The vast change in the motifs indicates the functional divergence of the GST with relevance to the variable substrates. Further, this identified motif and signature are group specific, which can be used as a query on the BLAST server for the identification of specific cyanobacterial GST groups (Supplementary Figure S2).

### 3.3. Evolutionary Divergence of Cyanobacterial GST

Phylogenetic analysis of 322 cyanobacterial GST for 126 cyanobacteria revealed five major clades and four minor clades (Figure 1). GST type Chi, cyGSTX1, cyGSTX2, and cyGSTX3, form separate major clades 1,2, 3, and 4 containing genus of the five orders except *Pleurocapsales,* indicating the absence of evolved forms of GSTs in primordial forms of cyanobacteria. cyGSTX4 in clade 10 is the only form of GST which is found in five orders of cyanobacteria. The hierarchal evolution of GST among cyanobacterial orders was evidenced by clade 8 (cyGSTX9) containing only the genus of primordial *Pleurocapsales* representing as an ancestor of all GSTs followed by clade 9 (cyGSTX6) which possesses the genus of primordial *Pleurocapsales,* lower order *Chroococcales,* and middle order *Oscillatoriales.* Clade 7 (cyGSTX7) contains only the genus of lower order *Chroococcales* and middle order *Oscillatoriales,* indicating this group has lost in primordial order *Pleurocapsales.* Clade 6 contains a cyGSTX5 representing the genus of *Pleurocapsales, Chroococcales* and *Nostocales* which suggests that this type might be lost in middle order *Oscillatoriales.* Clade 5 which is diverged into three minor clades cyGSTX8, cyGSTX10, and cyGSTX11 representing only the genus of *Nostocales*, diverged only in higher orders, which is lost in the lower, middle, and primordial orders. This proves that the GST evolved hierarchy from primordial *Pleurocapsales* to higher *Nostocales* substantiated the functional diversification of GSTs among the cyanobacteria forms.

GST families have diverse substrate specificity, which was acquired by the selection pressure during the course of evolution. Phylogenetic analysis revealed varying clustering of GST among the cyanobacterial orders (Figure 1). The distribution of GST among the cyanobacteria increases gradually from primordial *Pleurocapsales* to evolved *Nostocales* which clearly hints that the GSTs have undergone a series of selection pressure to craft a functionally divergent enzyme with high adaptability and significance. The average evolutionary distance among the five orders decreases from the lower unicellular forms to higher heterocystous forms which are 1.49 and 1.19 respectively (Table 3) suggesting that the primordial *Pleurocapsales* experienced a differential selection pressure which shaped the evolution of highly effective GSTs in higher order *Nostocales.* The low percentage of evolutionary divergence reducing from primordial order *Pleurocapsales* to higher order *Nostocale*s clearly evidenced the conserved nature of active residues.

### 3.4. Classification of GST Based on N-Terminal End, the G-Site

As the cyanobacterial GST sequences possess high sequence divergence, the identified 12 different GST classes were further analysed by the second level of grouping based on the distribution of the conserved signature residues present in the N-terminal end. The N-terminal end otherwise named as G-site adopts a thioredoxin like fold responsible for GSH substrate binding by providing a main chain donor and acceptor for GSH [28].

The two catalytic centres in the GST, GSH substrate binding site (G-site) and the hydrophobic substrate binding site (H-site) contribute a major role in assorting the classes. The G-site responsible for primary function is conserved among all classes. The H-site responsible for scavenging the variety of xenobiotic substrate facilitates functional diversification, hence it is less conserved. Catalytic residues at the G-site contribute major amino acids at active site, based on the amino acid participation in GSH binding, GST is sorted into two major classes, a Y-GST group which uses tyrosine and S/C-GST group which uses serine/cysteine to activate the GST at the N-terminal end [31].

**Figure 2:**
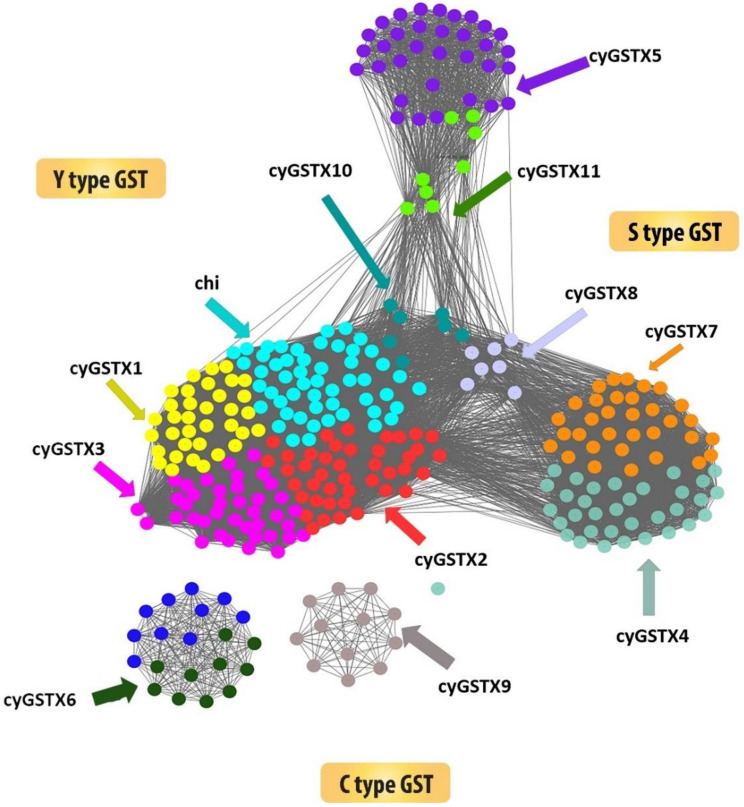
Showing the sequence similarity network of 322 cyanobacterial GST sequences. The network was thresholded at the BLAST E-Value of 1 × 10^−5^. Tyrosine types of GST were grouped separately from the diverse S/C type GSTs. For better understanding each node was coloured according to the GST types.

**Figure 3:**
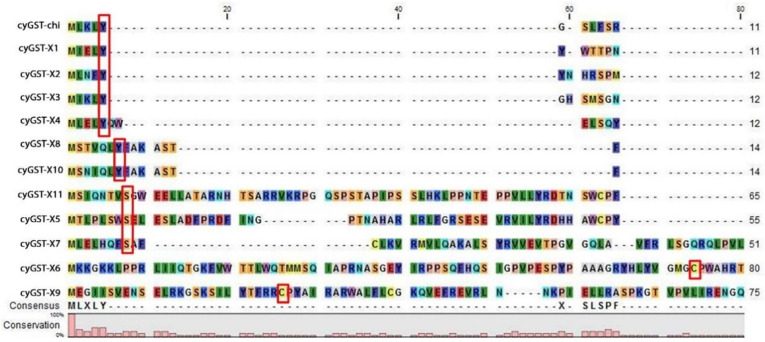
Sequence-based alignment of the representative cyanobacterial GST sequences showing the distribution of conserved signature residues cysteine, serine, and tyrosine highlighted in red boxes at the N-terminal end called the G-site. The alignment was prepared and displayed using the CLC bio-genomic workbench.

The structural backbone of Y-type and S/C type are found to be similar but the change in conserved residue at specific position within the fold has an important implication in the catalytic mechanism. The S/C type GST which uses its representative serine or cysteine residue is positioned at the amino terminus of the helix 1 to activate the bound GSH and serves as key to catalysis in most studied eukaryote and prokaryote enzymes [33]. Cyanobacterial GST X5, X6, X7, X9, and X11 have cysteine 26 or 74/serine 8 at the helix 1, but in some groups serine plays a critical function, despite the cysteine which is not required for the catalysis. Both cysteine and serine form a disulphide bond with GSH and transform the substrate. Some types have threonine in the position of S/C-GST and the threonine hydroxyl group forms a hydrogen bond with the sulfhydryl group of GSH. Cyanobacterial GSTX5, GSTX7, and GSTX11 have a reactive active residue serine which is replaced by cysteine in GSTX6 and GSTX9. The Y-type GST has tyrosine positioned at place 5 or 7 in the beta strand 1, the hydroxyl group in tyrosine serves as a hydrogen bond donor to the sulfur of GSH. The occurrence of active residue tyrosine at the beta strand 1 in the GST Chi, X1, X2, X3, X4, X8, and X10 designates it as Y-type (Figure 2). The most ancestral form of GST uses cysteine to activate the GSH, which change to serine in the middle-evolved forms and successful changes to tyrosine for catalysis [28]. Mapping of cyanobacterial GST onto the network leads to a new observation that has not been reported previously which also corroborated well with the phylogenetic tree. Sequence similarity poses strong evidence on separation of Y-type and S/C type of GSTs. The network fairly clusters all the GSTs in one, because it adopts a thioredoxin fold as a common domain. Figure 3 emphasizes the network drawn at the threshold e-value of 1 × 10^−10^, the network shows the multiple distribution of GSTs among the cyanobacteria. The distribution of the protein confirmed that Y type GSTs namely Chi, X1, X2, X3, X8, and X10 have grouped as a cluster, where C-type GSTs formed as a separate cluster. X9 and X6 are C-type GST that stood as a separate cluster. Likewise, the clustering of X5 and X11 belonging to S-type GST indicates the similarities, X4 is the only GST found in all orders clustered with X7 GST. These groupings represent the broad range of structural and functional diversity among the cyanobacteria. S-type GST is found in all orders of cyanobacteria, unlike C-type GST which was found in the lower and middle but absent in higher orders. Whereas Y-type GST is predominantly found in higher order *Nostocales*, followed by *Stigonematales*, *Chroococcales, and Oscillatoriales* suggesting the divergence of GST among the orders.

This grouping shares the perfect correspondence with the phylogeny. Among the cyanobacterial orders, primordial *Pleurocapsales* is generally considered to be the most ancestral has three S/C-group and one Y-group GSTs. Subsequently, three and two S/C-group GSTs were respectively found in middle evolved forms *Chroococcales* and *Oscillatoriales* along with five Y-groups GSTs in common, and the later evolved *Nostocales* having seven Y-groups and two S/C-groups GSTs. Similarly, *Stigonematales* possess only five Y-group GSTs. The largest diversity was found in *Nostocales,* and some of the classes were found to be lost in the other orders, because they became non-essential or evolution shaped them into more specific groups.

### 3.5. Structural Comparison of Cyanobacterial GST G-Site

Representative GST structures cyGSTX1, cyGSTX6, and cyGSTX7 which belong to the Y-group and S/C-group were analysed. Despite the high sequence differences, the backbone structure remains the same with two distinct domains: G-site at the N-terminal end and H-site at the C-terminal end. The N-terminal end adopts a topology similar to thioredoxin folds with βαβαββα_x_ structural arrangements and also has an alternative βαβα linked to the C-terminal end through the α-helices. Atkinson and Babbitt [31] reported the importance of tyrosine in Y-type and serine/cysteine in S/C- type and its location, implications for specificity differences between the classes of each subgroup. The sequence and structural analysis of all cyanobacterial GST types show that residues are found in two different locations. In Y-type, tyrosine is found in the β-strand 1, whereas in S/C- type the residue is found in the loop region before the α-helixes 1 at the N-terminal end. The degree of glutathione binding among these groups varies significantly which regulates the binding of substrate.

**Figure 4:**
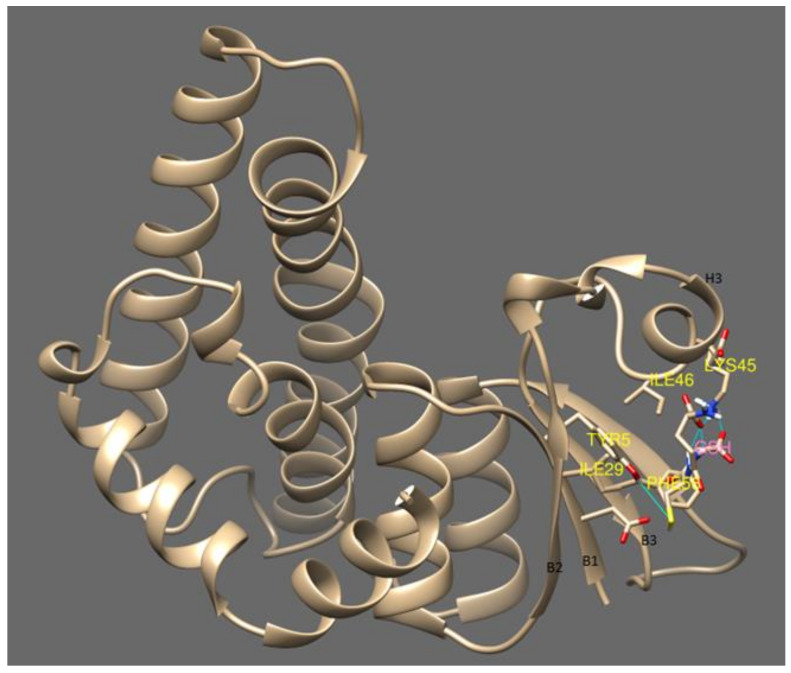
This shows the glutathione binding site with cyGSTX1 which belongs to the Y group. The active site for GSH binding involves beta strand 1, 2 and 3. Tyr 5 is found in the beta strand 1 and binds with the SH group of GSH. The active site was observed in the S and C type GST which is mentioned in Figure 5.

cyGSTX1 which belongs to the Y-group has the active site positioned among the cleft of the β- strands. GSH forms a non-covalent interaction with Tyr5, Ile29, Ile31, Gln40, Lys45, Il24, Phe56 which are located in the β-strands. The Tyr5 in β1 binds with the SH group of GSH, whereas Ile29, Ile31 from β2 form a hydrogen bond with the amine group of glutamine. Phe56 from β3 positions the GSH in the active site through Van der Waals interactions. Interaction of the OH group of Tyr5 not only activates the GSH, it also contributes to the stabilization by its interaction with α3 (Figure 4). The catalytic activity of active residue tyrosine investigated with site directed mutagenesis showed severe impairment in glutathione binding when replaced with His, Val or Thr [34]. A change in the topology of the active site will destabilize the interaction of GSH with GST resulting in different catalytic properties and signifying the importance of tyrosine in the Y-type GSTs.

**Figure 5:**
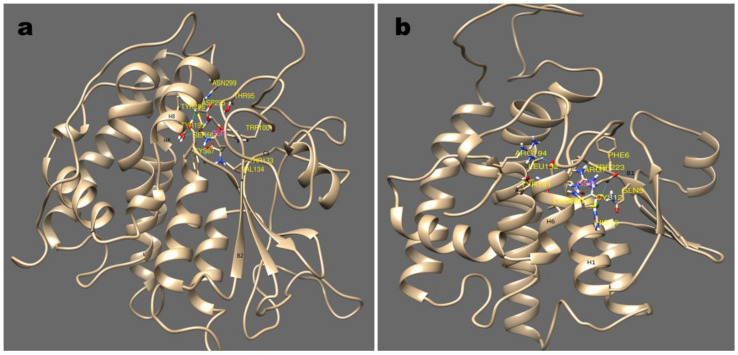
Major sub-groups of GST and its binding mode with substrate GSH are depicted in this picture (**a**); (**b**) show the stereo-view of GSH binding with cyGST X6 and X7, a representative structure for S and C GST. S type GST resides in GSH between helix 1,4, and 8 with the involvement of beta strand 2. Similarly with C type GST, the GSH interaction was observed with helix 1, and 7 and beta strand 1.

Differently, in the S/C type the active site is shifted in between the helix which is entirely different from Y-type. cyGSTX7, belonging to S-type, positions the GSH between the α-helix 1, 4, and 8. Ser66 which is found in α-helix 1 interacts with the SH group of GSH, the interaction is further stabilized by α-helix 4 and 8, which forms an N-terminal domains (Figure 5a). Caccuri et al. [35] reported in *Lucilia cuprina* GST, the mutation of Ser to Ala has less than 0.5% of normal activity which shows weak affinity towards glutathione. Similarly, in cyGSTX6 a C-type group, GSH is found in Trx domain, Cys12 present in α-helix 1 interacts with the SH group of GSH. The interaction is stabilized by the involvement of α-helix 6 and 7 (Figure 5b). In human GST P Cys14 is reported to participate in the catalytic reaction of GST by stabilizing the conformation of the active-site loop, but not in the GSH, binding directly [36]. Involvement of α-helix in GSH binding remains the same for both S and C type GST groups, but the active site shift was observed in the Y-type group indicating the ubiquity and diversity of GSTs among the cyanobacterial orders. The commonalities among the cyanobacterial GST groups clearly indicate that GST has evolved by means of convergent evolution, the thioredoxin folds provide the foundation for all forms of GSTs. The active site shift occurring in the recently evolved Y-type might enhance the catalytic activities with significance for evolutionarily conserved Tyr7.

## 4. Conclusions

It was observed that cyanobacterial GST, an important class of detoxification enzyme, has evolved in hierarchy and might possess a divergent functional role. This study highlights the distribution of the 12 GST classes among the cyanobacteria such as primordial order *Pleurocapsales* with four GST isoforms which serves as an ancestor, *Chroococcales* with eight classes in lower order, seven in middle order *Oscillatoriales*, five in *Stigonematales*, and nine in higher order *Nostocale*s. Despite its high diversity, the evolutionary distance evidenced the retaining of the ancestral GSTs function in all cyanobacterial orders. All cyanobacterial GSTs having the backbone of thioredoxin fold are categorized into two groups namely Y-GST and S/C-GSTs. *Pleurocapsales* with a simple photosynthetic apparatus possess only four GST classes which are one Y-type and three S/C-type GST. The well-organized *Chroococcales* and filamentous forms *Oscillatoriales* have eight and seven classes of GST comprising five Y-type GST, three S-type, and two C-type GST groups. While, the order *Stigonematales* has five GST classes all comprising the Y-type GST group. On the other hand, heterocystous *Nostocales* possesses nine classes grouped into seven Y-type and two S-type GST groups. The shifted active site of Y-GST away from α1 might enhance the catalytic capabilities compared to S/C-GSTs. The distinctive characteristics of analysed GSTs evidence that recently evolved order *Nostocales* with higher Y-GST classes might possess more active GSTs which are more evolutionary divergent compared to the lower and middle order cyanobacteria.

## Figures and Tables

**Table 1:** Diversity and the distribution of glutathione S-transferases (GST) types in five orders of cyanobacteria.

Orders	Chi	cyGSTX1	cyGSTX2	cyGSTX3	cyGSTX4	cyGSTX5	cyGSTX6	cyGSTX7	cyGSTX8	cyGSTX9	cyGSTX10	cyGSTX11
*Pleurocapsales*	✕	✕	✕	✕	✓	✓	✓	✕	✕	✓	✕	✕
*Chroococcales*	✓	✓	✓	✓	✓	✓	✓	✓	✕	✕	✕	✕
*Oscillatoriales*	✓	✓	✓	✓	✓	✕	✓	✓	✕	✕	✕	✕
*Nostocales*	✓	✓	✓	✓	✓	✓	✕	✕	✓	✕	✓	✓
*Stigonematales*	✓	✓	✓	✓	✓	✕	✕	✕	✕	✕	✕	✕

**Table 2:** Type specific conserved motifs unique to specific types of the cyanobacterial GSTs.

Orders	Chi	cyGSTX1	cyGSTX2	cyGSTX3	cyGSTX4	cyGSTX5	cyGSTX6	cyGSTX7	cyGSTX8	cyGSTX9	cyGSTX10	cyGSTX11
*Pleurocapsales*	✕	✕	✕	✕	STEIA	SDDI(IL)	SAEII	✕	✕	SL(ED)I(IM)	✕	✕
*Chroococcales*	SGAIL	SGAIL	SLAIL	SNA(IV)L	ST(EDA)IA	SD(DRV)II	SAEII	S(ST)AI(AC)	✕	✕	✕	✕
*Oscillatoriales*	SGAIL	SGAIL	S(FL)AIL	SNA(IM)(LM)	ST(AE)IA	✕	SA(DE)II	SSAIA	✕	✕	✕	✕
*Nostocales*	SGAIL	SGAIL	SLAI(LM)	SNAIL	ST(EA)IA	SDDII	✕	✕	SAI(IV)N	✕	SA(IV)IN	SKDIL
*Stigonematales*	SGAIL	SGAIL	SLAIL	SNAIL	STEIA	✕	✕	✕	✕	✕	✕	✕
*Motif architecture for each GST class*	SGAIL	SGAIL	S(FL)AI(LM)	SNA(IVM)(LM)	ST(EDA)IA	SD(DRV)I(IL)	SA(DE)II	S(ST)AI(AC)	SAI(IV)N	SL(ED)I(IM)	SA(IV)IN	SKDIL

**Table 3:** Shows the evolutionary divergence in percent over amino acid sequences between each order.

	*Chroococcales*	*Pleurocapsales*	*Oscillatoriales*	*Nostocales*	*Stigonematales*
*Chroococcales*		0.0619	0.0655	0.0577	0.0609
*Pleurocapsales*	1.4035		0.0681	0.0598	0.0637
*Oscillatoriales*	1.3594	1.4166		0.0634	0.0664
*Nostocales*	1.3054	1.3361	1.3376		0.0582
*Stigonematales*	1.2731	1.3103	1.2907	1.1904	

## Supplementary Materials

The following are available online at https://www.mdpi.com/2076-2607/8/5/712/s1, Figure S1: Multiple sequence alignment of 322 GST sequence found in 126 cyanobacterial sequences showing the GST motif architecture of “ES [GLNTARS][ADE]I[LAI]”. The conserved domain is highlighted in the pink box. Sequence alignment was performed as mentioned in Section 2.2., Figure S2: Highlight of the 12 different GST motif structures identified in 322 retrieved GST sequences belonging to five cyanobacterial orders. Sequence belonging to each clade was removed manually and sequence alignment performed as mentioned in Section 2.2. The sequence logo was generated by subjecting the aligned file to Weblogo online server [13]. Table S1: Detailed information of the cyanobacterial GST sequences used for the construction of phylogenetic tree.

## References

[1] Wiktelius E., Stenberg G. (2007). Novel class of glutathione transferases from cyanobacteria exhibit high catalytic activities towards naturally occurring isothiocyanates. Biochem. J..

[2] Jerina D.M., Bend J.R. (1977). Glutathione S-transferases. Biological Reactive Intermediates.

[3] Apel K., Hirt H. (2004). Reactive oxygen species: Metabolism, oxidative stress, and signal transduction. Annu. Rev. Plant. Biol..

[4] Oakley A. (2011). Glutathione transferases: A structural perspective. Drug Metab. Rev..

[5] Rossjohn J., McKinstry W.J., Oakley A.J., Verger D., Flanagan J., Chelvanayagam G., Parker M.W. (1998). Human theta class glutathione transferase: The crystal structure reveals a sulfate-binding pocket within a buried active site. Structure.

[6] Hayes J.D., Flanagan J.U., Jowsey I.R. (2005). Glutathione transferases. Annu. Rev. Pharmacol. Toxicol..

[7] Sherratt P.J., Hayes J.D. (2001). Glutathione S-transferases. Enzyme Systems That Metabolise Drugs and Other Xenobiotics.

[8] Frova C. (2006). Glutathione transferases in the genomics era: New insights and perspectives. Biomol. Eng..

[9] Sheehan D., Meade G., Foley V., Dowd C. (2001). Structure, function and evolution of glutathione transferases: Implications for classification of non-mammalian members of an ancient enzyme superfamily. Biochem. J..

[10] Blanchette B., Feng X., Singh B.R. (2007). Marine glutathione S-transferases. Mar. Biotechnol..

[11] Allocati N., Federici L., Masulli M., Di Ilio C. (2009). Glutathione transferases in bacteria. Febs J..

[12] Pandey T., Shukla R., Shukla H., Sonkar A., Tripathi T., Singh A.K. (2017). A combined biochemical and computational studies of the rho-class glutathione s-transferase sll1545 of synechocystis PCC 6803. Int. J. Biol. Macromol..

[13] Crooks G.E., Hon G., Chandonia J.M., Brenner S.E. (2004). WebLogo: A sequence logo generator. Genome. Res..

[14] Dixon D.P., Lapthorn A., Edwards R. (2002). Plant glutathione transferases. Genome Biol..

[15] Cameron J.C., Pakrasi H.B. (2010). Essential role of glutathione in acclimation to environmental and redox perturbations in the cyanobacterium *Synechocystis* sp. PCC 6803. Plant. Physiol..

[16] Shylajanaciyar M., Dineshbabu G., Rajalakshmi R., Subramanian G., Prabaharan D., Uma L. (2015). Analysis and elucidation of phosphoenolpyruvate carboxylase in cyanobacteria. Protein J..

[17] Shih P.M., Wu D., Latifi A., Axen S.D., Fewer D.P., Talla E., Kerfeld C.A. (2013). Improving the coverage of the cyanobacterial phylum using diversity-driven genome sequencing. Proc. Natl. Acad. Sci. USA.

[18] Peter A.P., Lakshmanan K., Mohandass S., Varadharaj S., Thilagar S., Kareem K.A.A., Prabaharan D., Gopalakrishnan S., Lakshmanan U. (2015). Cyanobacterial knowledge base (CKB), a compendium of cyanobacterial genomes and proteomes. PLoS ONE.

[19] Katoh K., Standley D.M. (2013). MAFFT multiple sequence alignment software version 7: Improvements in performance and usability. Mol. Biol. Evol..

[20] Capella-Gutierrez S., Silla-Martinez J.M., Gabaldon T. (2009). Trimal: A tool for automated alignment trimming in large-scale phylogenetic analyses. Bioinformatics.

[21] Tamura K., Peterson D., Peterson N., Stecher G., Nei M., Kumar S. (2011). MEGA5: Molecular evolutionary genetics analysis using maximum likelihood, evolutionary distance, and maximum parsimony methods. Mol. Biol. Evol..

[22] Rambaut A. (2009). FigTree v1. 3.1: Tree Figure Drawing Tool. http://tree.bio.ed.ac.uk/software/figtree/.

[23] Skopelitou K., Dhavala P., Papageorgiou A.C., Labrou N.E. (2012). A glutathione transferase from *Agrobacterium tumefaciens* reveals a novel class of bacterial GST superfamily. PLoS ONE.

[24] Gerlt J.A., Bouvier J.T., Davidson D.B., Imker H.J., Sadkhin B., Slater D.R., Whalen K.L. (2015). Enzyme function initiative-enzyme similarity tool (EFI-EST): A web tool for generating protein sequence similarity networks. BBA Proteins Proteom..

[25] Shannon P., Markiel A., Ozier O., Baliga N.S., Wang J.T., Ramage D., Amin N., Schwikowski B., Ideker T. (2003). Cytoscape: A software environment for integrated models of biomolecular interaction networks. Genome Res..

[26] Morris G.M., Huey R., Lindstrom W., Sanner M.F., Belew R.K., Goodsell D.S., Olson A.J. (2009). Autodock4 and AutoDockTools4: Automated docking with selective receptor flexibility. J. Comput. Chem..

[27] Fuhrmann J., Rurainski A., Lenhof H.P., Neumann D. (2010). A new Lamarckian genetic algorithm for flexible ligand receptor docking. J. Comput. Chem..

[28] Da Fonseca R.R., Johnson W.E., O’Brien S.J., Vasconcelos V., Antunes A. (2010). Molecular evolution and the role of oxidative stress in the expansion and functional diversification of cytosolic glutathione transferases. BMC Evol. Biol..

[29] Pandey T., Singh S.K., Chhetri G., Tripathi T., Singh A.K. (2015). Characterization of a highly pH stable chi-class glutathione S-transferase from synechocystis PCC 6803. PLoS ONE.

[30] Eaton D.L., Bammler T.K. (1999). Concise review of the glutathione S-transferases and their significance to toxicology. Toxicol. Sci..

[31] Atkinson H.J., Babbitt P.C. (2009). Glutathione transferases are structural and functional outliers in the thioredoxin fold. Biochemistry.

[32] Singh P., Kaushik M.S., Srivastava M., Mishra A.K. (2014). Phylogenetic analysis of heterocystous cyanobacteria (subsections IV and V) using highly iterated palindromes as molecular markers. Physiol. Mol. Biol. Plants.

[33] Board P.G., Coggan M., Chelvanayagam G., Easteal S., Jermiin L.S., Schulte G.K., Danley D.E., Hoth L.R., Griffor M.C., Kamath A.V. (2000). Identification, characterization, and crystal structure of the Omega class glutathione transferases. J. Biol. Chem..

[34] Dirr H., Reinemer P., Huber R. (1994). X-ray crystal structures of cytosolic glutathione S-transferases. Eur. J. Biochem..

[35] Caccuri A.M., Antonini G., Nicotra M., Battistoni A., Bello M.L., Board P.G., Parker M.W., Ricci G. (1997). Catalytic mechanism and role of hydroxyl residues in the active site of theta class glutathione S-transferases. Investigation of Ser-9 and Tyr-113 in a glutathione S-transferase from the Australian sheep blowfly, *Lucilia cuprina*. J. Biol. Chem..

[36] Park H.J., Lee G.S., Gong G.H. (2001). Functional studies of cysteine residues in human glutathione S-transferase P1-1 by site-directed mutagenesis. Bull. Korean Chem. Soc..

