# Supplementary material for: Diversity of Glutathione S-Transferases (GSTs) in Cyanobacteria with Reference to Their Structures, Substrate Recognition and Catalytic Functions
Source: Microorganisms. 2020 May 11;8(5):712. doi: 10.3390/microorganisms8050712 (PMC7286025; doi:10.3390/microorganisms8050712)
Supplement: Supplementary file 1 [file microorganisms-08-00712-s001.zip › microorganisms-740137-supplementary.pdf]

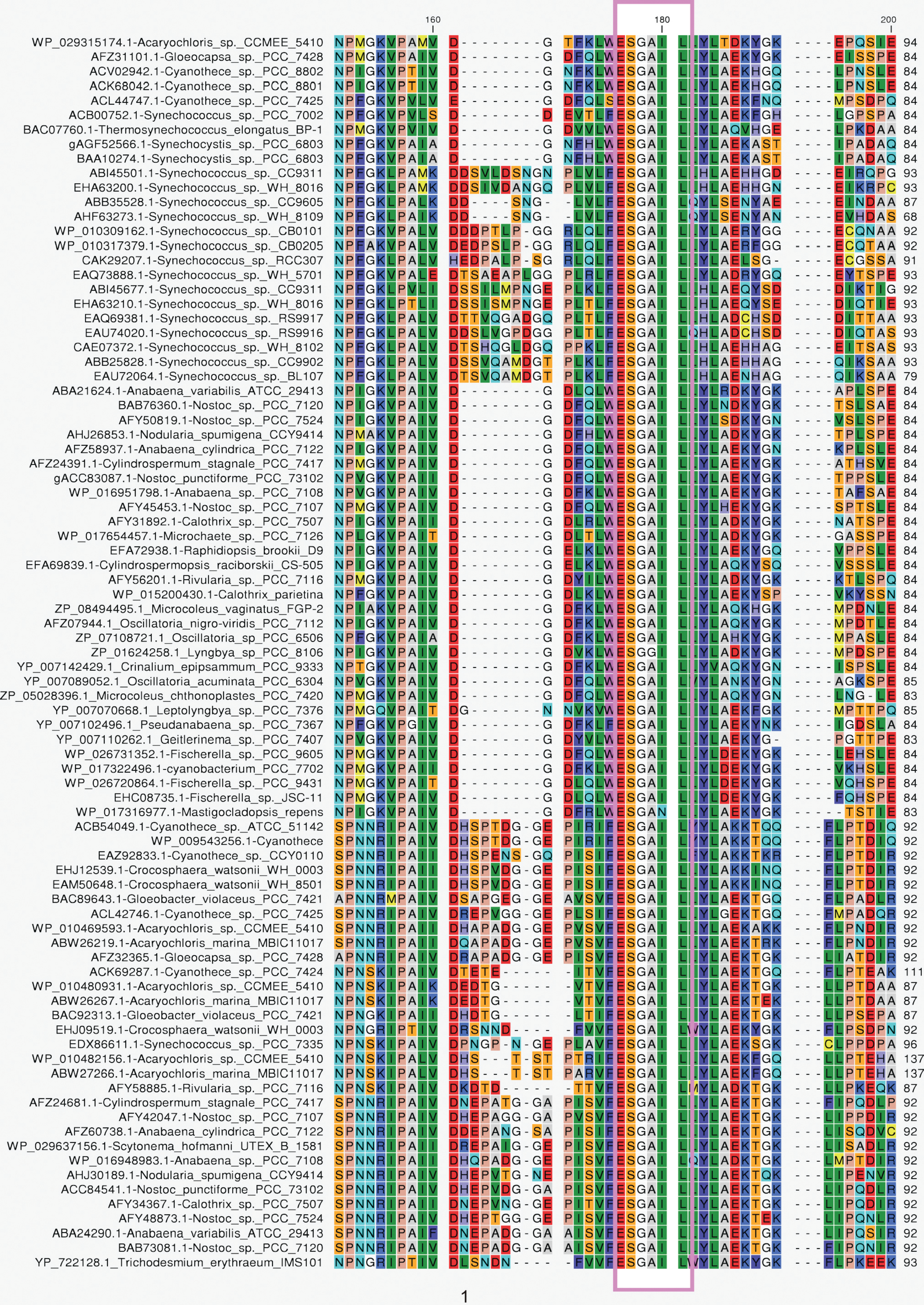
160
180
200
WP_029315174.1-Acaryochloris_sp._CCMEE_5410 94
AFZ31101.1-Gloeocapsa_sp._PCC_7428 84
ACV02942.1-Cyanothece_sp._PCC_8802 84
ACK68042.1-Cyanothece_sp._PCC_8801 84
ACL44747.1-Cyanothece_sp._PCC_7425 84
ACB00752.1-Synechococcus_sp._PCC_7002 84
BAC07760.1-Thermosynechococcus_elongatus_BP-1 84
gAGF52566.1-Synechocystis_sp._PCC_6803 84
BAA10274.1-Synechocystis_sp._PCC_6803 84
ABI45501.1-Synechococcus_sp._CC9311 93
EHA63200.1-Synechococcus_sp._WH_8016 93
ABB35528.1-Synechococcus_sp._CC9605 87
AHF63273.1-Synechococcus_sp._WH_8109 68
WP_010309162.1-Synechococcus_sp._CB0101 92
WP_010317379.1-Synechococcus_sp._CB0205 92
CAK29207.1-Synechococcus_sp._RCC307 91
EAQ73888.1-Synechococcus_sp._WH_5701 93
ABI45677.1-Synechococcus_sp._CC9311 92
EHA63210.1-Synechococcus_sp._WH_8016 93
EAQ69381.1-Synechococcus_sp._RS9917 93
EAU74020.1-Synechococcus_sp._RS9916 93
CAE07372.1-Synechococcus_sp._WH_8102 93
ABB25828.1-Synechococcus_sp._CC9902 93
EAU72064.1-Synechococcus_sp._BL107 79
ABA21624.1-Anabaena_variabilis_ATCC_29413 84
BAB76360.1-Nostoc_sp._PCC_7120 84
AFY50819.1-Nostoc_sp._PCC_7524 84
AHJ26853.1-Nodularia_spumigena_CCY9414 84
AFZ58937.1-Anabaena_cylindrica_PCC_7122 84
AFZ24391.1-Cylindrospermum_stagnale_PCC_7417 84
gACC83087.1-Nostoc_punctiforme_PCC_73102 84
WP_016951798.1-Anabaena_sp._PCC_7108 84
AFY45453.1-Nostoc_sp._PCC_7107 84
AFY31892.1-Calothrix_sp._PCC_7507 84
WP_017654457.1-Microchaete_sp._PCC_7126 84
EFA72938.1-Raphidiopsis_brookii_D9 84
EFA69839.1-Cylindrospermopsis_raciborskii_CS-505 84
AFY56201.1-Rivularia_sp._PCC_7116 84
WP_015200430.1-Calothrix_parietina 84
ZP_08494495.1_Microcoleus_vaginatus_FGP-2 84
AFZ07944.1_Oscillatoria_nigro-viridis_PCC_7112 84
ZP_07108721.1_Oscillatoria_sp_PCC_6506 84
ZP_01624258.1_Lyngbya_sp_PCC_8106 84
YP_007142429.1_Crinalium_epipsammum_PCC_9333 84
YP_007089052.1_Oscillatoria_acuminata_PCC_6304 85
ZP_05028396.1_Microcoleus_chthonoplastes_PCC_7420 83
YP_007070668.1_Leptolyngbya_sp._PCC_7376 85
YP_007102496.1_Pseudanabaena_sp._PCC_7367 84
YP_007110262.1_Geitlerinema_sp._PCC_7407 83
WP_026731352.1-Fischerella_sp._PCC_9605 84
WP_017322496.1-cyanobacterium_PCC_7702 84
WP_026720864.1-Fischerella_sp._PCC_9431 84
EHC08735.1-Fischerella_sp._JSC-11 84
WP_017316977.1-Mastigocladopsis_repens 83
ACB54049.1-Cyanothece_sp._ATCC_51142 92
WP_009543256.1-Cyanothece 92
EAZ92833.1-Cyanothece_sp._CCY0110 92
EHJ12539.1-Crocosphaera_watsonii_WH_0003 92
EAM50648.1-Crocosphaera_watsonii_WH_8501 92
BAC89643.1-Gloeobacter_violaceus_PCC_7421 92
ACL42746.1-Cyanothece_sp._PCC_7425 92
WP_010469593.1-Acaryochloris_sp._CCMEE_5410 92
ABW26219.1-Acaryochloris_marina_MBIC11017 92
AFZ32365.1-Gloeocapsa_sp._PCC_7428 92
ACK69287.1-Cyanothece_sp._PCC_7424 111
WP_010480931.1-Acaryochloris_sp._CCMEE_5410 87
ABW26267.1-Acaryochloris_marina_MBIC11017 87
BAC92313.1-Gloeobacter_violaceus_PCC_7421 87
EHJ09519.1-Crocosphaera_watsonii_WH_0003 92
EDX86611.1-Synechococcus_sp._PCC_7335 96
WP_010482156.1-Acaryochloris_sp._CCMEE_5410 137
ABW27266.1-Acaryochloris_marina_MBIC11017 137
AFY58885.1-Rivularia_sp._PCC_7116 87
AFZ24681.1-Cylindrospermum_stagnale_PCC_7417 92
AFY42047.1-Nostoc_sp._PCC_7107 92
AFZ60738.1-Anabaena_cylindrica_PCC_7122 92
WP_029637156.1-Scytonema_hofmanni_UTEX_B_1581 92
WP_016948983.1-Anabaena_sp._PCC_7108 92
AHJ30189.1-Nodularia_spumigena_CCY9414 92
ACC84541.1-Nostoc_punctiforme_PCC_73102 92
AFY34367.1-Calothrix_sp._PCC_7507 92
AFY48873.1-Nostoc_sp._PCC_7524 92
ABA24290.1-Anabaena_variabilis_ATCC_29413 92
BAB73081.1-Nostoc_sp._PCC_7120 92
YP_722128.1_Trichodesmium_erythraeum_IMS101 93

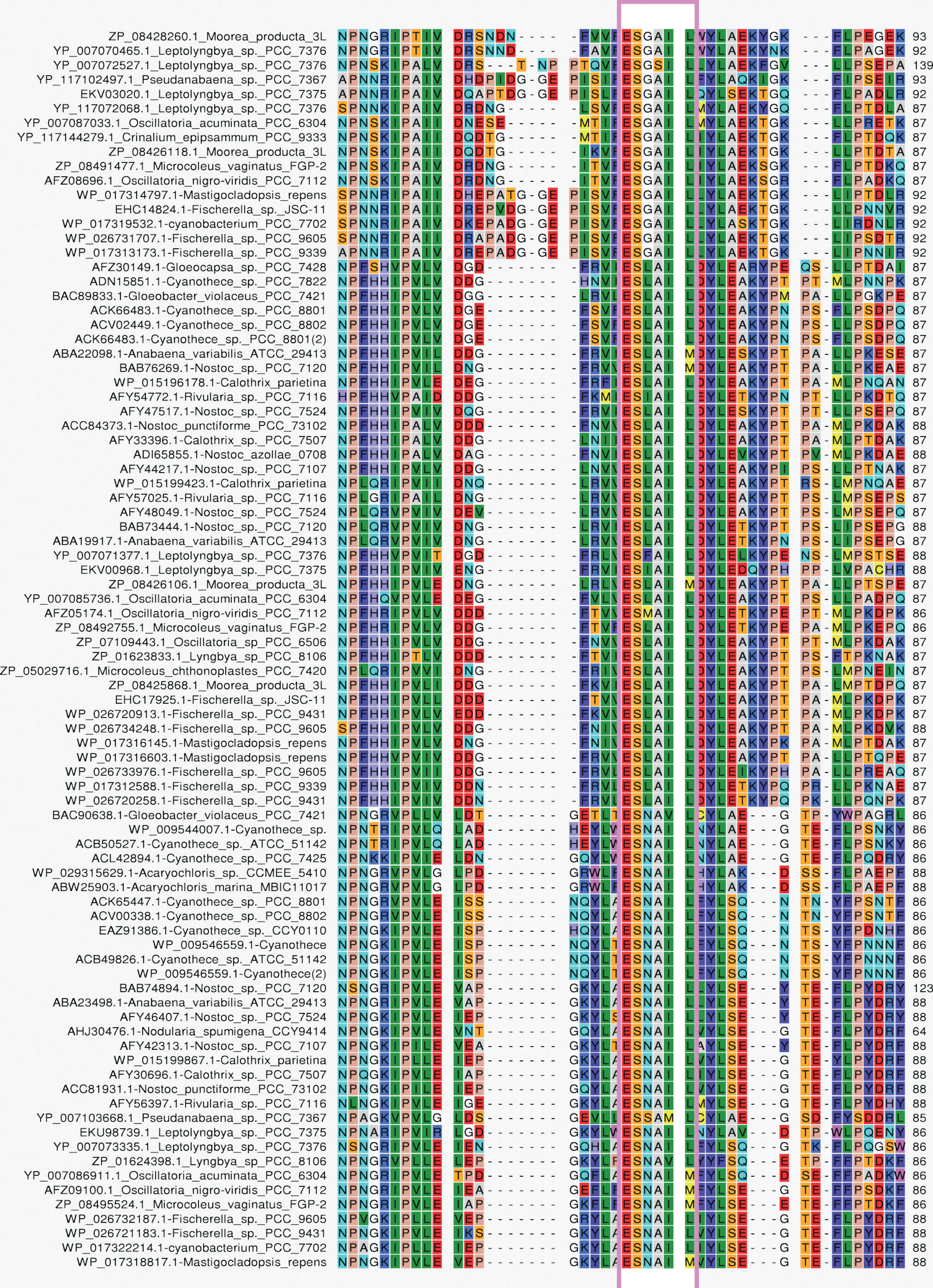

ZP_08428260.1_Moorea_producta_3L 93
YP_007070465.1_Leptolyngbya_sp._PCC_7376 92
YP_007072527.1_Leptolyngbya_sp._PCC_7376 139
YP_117102497.1_Pseudanabaena_sp._PCC_7367 93
EKV03020.1_Leptolyngbya_sp._PCC_7375 92
YP_117072068.1_Leptolyngbya_sp._PCC_7376 87
YP_007087033.1_Oscillatoria_acuminata_PCC_6304 87
YP_117144279.1_Crinalium_epipsammum_PCC_9333 87
ZP_08426118.1_Moorea_producta_3L 87
ZP_08491477.1_Microcoleus_vaginatus_FGP-2 87
AFZ08696.1_Oscillatoria_nigro-viridis_PCC_7112 87
WP_017314797.1-Mastigocladopsis_repens 92
EHC14824.1-Fischerella_sp._JSC-11 92
WP_017319532.1-cyanobacterium_PCC_7702 92
WP_026731707.1-Fischerella_sp._PCC_9605 92
WP_017313173.1-Fischerella_sp._PCC_9339 92
AFZ30149.1-Gloeocapsa_sp._PCC_7428 87
ADN15851.1-Cyanothece_sp._PCC_7822 87
BAC89833.1-Gloeobacter_violaceus_PCC_7421 87
ACK66483.1-Cyanothece_sp._PCC_8801 87
ACV02449.1-Cyanothece_sp._PCC_8802 87
ACK66483.1-Cyanothece_sp._PCC_8801(2) 87
ABA22098.1-Anabaena_variabilis_ATCC_29413 87
BAB76269.1-Nostoc_sp._PCC_7120 87
WP_015196178.1-Calothrix_parietina 87
AFY54772.1-Rivularia_sp._PCC_7116 87
AFY47517.1-Nostoc_sp._PCC_7524 87
ACC84373.1-Nostoc_punctiforme_PCC_73102 88
AFY33396.1-Calothrix_sp._PCC_7507 87
ADI65855.1-Nostoc_azollae_0708 88
AFY44217.1-Nostoc_sp._PCC_7107 87
WP_015199423.1-Calothrix_parietina 87
AFY57025.1-Rivularia_sp._PCC_7116 87
AFY48049.1-Nostoc_sp._PCC_7524 87
BAB73444.1-Nostoc_sp._PCC_7120 88
ABA19917.1-Anabaena_variabilis_ATCC_29413 87
YP_007071377.1_Leptolyngbya_sp._PCC_7376 88
EKV00968.1_Leptolyngbya_sp._PCC_7375 88
ZP_08426106.1_Moorea_producta_3L 87
YP_007085736.1_Oscillatoria_acuminata_PCC_6304 87
AFZ05174.1_Oscillatoria_nigro-viridis_PCC_7112 86
ZP_08492755.1_Microcoleus_vaginatus_FGP-2 86
ZP_07109443.1_Oscillatoria_sp_PCC_6506 87
ZP_01623833.1_Lyngbya_sp_PCC_8106 87
ZP_05029716.1_Microcoleus_chthonoplastes_PCC_7420 87
ZP_08425868.1_Moorea_producta_3L 87
EHC17925.1-Fischerella_sp._JSC-11 87
WP_026720913.1-Fischerella_sp._PCC_9431 87
WP_026734248.1-Fischerella_sp._PCC_9605 88
WP_017316145.1-Mastigocladopsis_repens 87
WP_017316603.1-Mastigocladopsis_repens 87
WP_026733976.1-Fischerella_sp._PCC_9605 87
WP_017312588.1-Fischerella_sp._PCC_9339 87
WP_026720258.1-Fischerella_sp._PCC_9431 87
BAC90638.1-Gloeobacter_violaceus_PCC_7421 86
WP_009544007.1-Cyanothece_sp. 86
ACB50527.1-Cyanothece_sp._ATCC_51142 86
ACL42894.1-Cyanothece_sp._PCC_7425 86
WP_029315629.1-Acaryochloris_sp._CCMEE_5410 88
ABW25903.1-Acaryochloris_marina_MBIC11017 88
ACK65447.1-Cyanothece_sp._PCC_8801 86
ACV00338.1-Cyanothece_sp._PCC_8802 86
EAZ91386.1-Cyanothece_sp._CCY0110 86
WP_009546559.1-Cyanothece 86
ACB49826.1-Cyanothece_sp._ATCC_51142 86
WP_009546559.1-Cyanothece(2) 86
BAB74894.1-Nostoc_sp._PCC_7120 123
ABA23498.1-Anabaena_variabilis_ATCC_29413 88
AFY46407.1-Nostoc_sp._PCC_7524 88
AHJ30476.1-Nodularia_spumigena_CCY9414 64
AFY42313.1-Nostoc_sp._PCC_7107 88
WP_015199867.1-Calothrix_parietina 88
AFY30696.1-Calothrix_sp._PCC_7507 88
ACC81931.1-Nostoc_punctiforme_PCC_73102 88
AFY56397.1-Rivularia_sp._PCC_7116 88
YP_007103668.1_Pseudanabaena_sp._PCC_7367 85
EKU98739.1_Leptolyngbya_sp._PCC_7375 86
YP_007073335.1_Leptolyngbya_sp._PCC_7376 86
ZP_01624398.1_Lyngbya_sp_PCC_8106 86
YP_007086911.1_Oscillatoria_acuminata_PCC_6304 86
AFZ09100.1_Oscillatoria_nigro-viridis_PCC_7112 86
ZP_08495524.1_Microcoleus_vaginatus_FGP-2 86
WP_026732187.1-Fischerella_sp._PCC_9605 88
WP_026721183.1-Fischerella_sp._PCC_9431 88
WP_017322214.1-cyanobacterium_PCC_7702 88
WP_017318817.1-Mastigocladopsis_repens 88

2

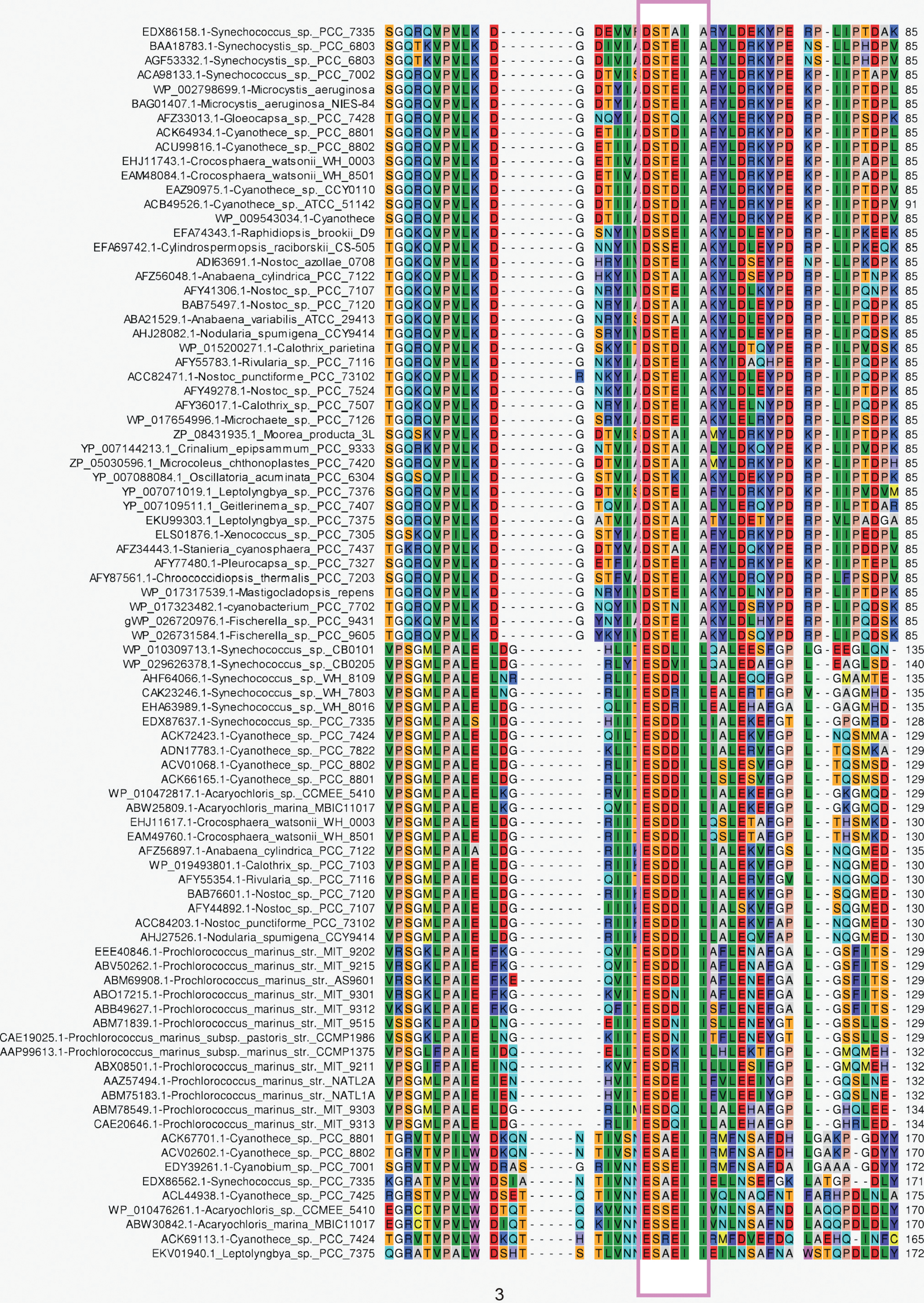
EDX86158.1-Synechococcus_sp._PCC_7335 85
BAA18783.1-Synechocystis_sp._PCC_6803 85
AGF53332.1-Synechocystis_sp._PCC_6803 85
ACA98133.1-Synechococcus_sp._PCC_7002 85
WP_002798699.1-Microcystis_aeruginosa 85
BAG01407.1-Microcystis_aeruginosa_NIES-84 85
AFZ33013.1-Gloeocapsa_sp._PCC_7428 85
ACK64934.1-Cyanothece_sp._PCC_8801 85
ACU99816.1-Cyanothece_sp._PCC_8802 85
EHJ11743.1-Crocosphaera_watsonii_WH_0003 85
EAM48084.1-Crocosphaera_watsonii_WH_8501 85
EAZ90975.1-Cyanothece_sp._CCY0110 85
ACB49526.1-Cyanothece_sp._ATCC_51142 91
WP_009543034.1-Cyanothece 85
EFA74343.1-Raphidiopsis_brookii_D9 85
EFA69742.1-Cylindrospermopsis_raciborskii_CS-505 85
ADI63691.1-Nostoc_azollae_0708 85
AFZ56048.1-Anabaena_cylindrica_PCC_7122 85
AFY41306.1-Nostoc_sp._PCC_7107 85
BAB75497.1-Nostoc_sp._PCC_7120 85
ABA21529.1-Anabaena_variabilis_ATCC_29413 85
AHJ28082.1-Nodularia_spumigena_CCY9414 85
WP_015200271.1-Calothrix_parietina 85
AFY55783.1-Rivularia_sp._PCC_7116 85
ACC82471.1-Nostoc_punctiforme_PCC_73102 85
AFY49278.1-Nostoc_sp._PCC_7524 85
AFY36017.1-Calothrix_sp._PCC_7507 85
WP_017654996.1-Microchaete_sp._PCC_7126 85
ZP_08431935.1_Moorea_producta_3L 85
YP_007144213.1_Crinalium_epipsammum_PCC_9333 85
ZP_05030596.1_Microcoleus_chthonoplastes_PCC_7420 85
YP_007088084.1_Oscillatoria_acuminata_PCC_6304 85
YP_007071019.1_Leptolyngbya_sp._PCC_7376 85
YP_007109511.1_Geitlerinema_sp._PCC_7407 85
EKU99303.1_Leptolyngbya_sp._PCC_7375 85
ELS01876.1-Xenococcus_sp._PCC_7305 85
AFZ34443.1-Stanieria_cyanosphaera_PCC_7437 85
AFY77480.1-Pleurocapsa_sp._PCC_7327 85
AFY87561.1-Chroococcidiopsis_thermalis_PCC_7203 85
WP_017317539.1-Mastigocladopsis_repens 85
WP_017323482.1-cyanobacterium_PCC_7702 85
gWP_026720976.1-Fischerella_sp._PCC_9431 85
WP_026731584.1-Fischerella_sp._PCC_9605 85
WP_010309713.1-Synechococcus_sp._CB0101 135
WP_029626378.1-Synechococcus_sp._CB0205 140
AHF64066.1-Synechococcus_sp._WH_8109 135
CAK23246.1-Synechococcus_sp._WH_7803 135
EHA63989.1-Synechococcus_sp._WH_8016 135
EDX87637.1-Synechococcus_sp._PCC_7335 128
ACK72423.1-Cyanothece_sp._PCC_7424 129
ADN17783.1-Cyanothece_sp._PCC_7822 129
ACV01068.1-Cyanothece_sp._PCC_8802 129
ACK66165.1-Cyanothece_sp._PCC_8801 129
WP_010472817.1-Acaryochloris_sp._CCMEE_5410 129
ABW25809.1-Acaryochloris_marina_MBIC11017 129
EHJ11617.1-Crocosphaera_watsonii_WH_0003 130
EAM49760.1-Crocosphaera_watsonii_WH_8501 130
AFZ56897.1-Anabaena_cylindrica_PCC_7122 135
WP_019493801.1-Calothrix_sp._PCC_7103 130
AFY55354.1-Rivularia_sp._PCC_7116 130
BAB76601.1-Nostoc_sp._PCC_7120 130
AFY44892.1-Nostoc_sp._PCC_7107 130
ACC84203.1-Nostoc_punctiforme_PCC_73102 130
AHJ27526.1-Nodularia_spumigena_CCY9414 130
EEE40846.1-Prochlorococcus_marinus_str._MIT_9202 129
ABV50262.1-Prochlorococcus_marinus_str._MIT_9215 129
ABM69908.1-Prochlorococcus_marinus_str._AS9601 129
ABO17215.1-Prochlorococcus_marinus_str._MIT_9301 129
ABB49627.1-Prochlorococcus_marinus_str._MIT_9312 129
ABM71839.1-Prochlorococcus_marinus_str._MIT_9515 129
CAE19025.1-Prochlorococcus_marinus_subsp._pastoris_str._CCMP1986 129
AAP99613.1-Prochlorococcus_marinus_subsp._marinus_str._CCMP1375 132
ABX08501.1-Prochlorococcus_marinus_str._MIT_9211 132
AAZ57494.1-Prochlorococcus_marinus_str._NATL2A 132
ABM75183.1-Prochlorococcus_marinus_str._NATL1A 132
ABM78549.1-Prochlorococcus_marinus_str._MIT_9303 134
CAE20646.1-Prochlorococcus_marinus_str._MIT_9313 134
ACK67701.1-Cyanothece_sp._PCC_8801 170
ACV02602.1-Cyanothece_sp._PCC_8802 170
EDY39261.1-Cyanobium_sp._PCC_7001 172
EDX86562.1-Synechococcus_sp._PCC_7335 171
ACL44938.1-Cyanothece_sp._PCC_7425 175
WP_010476261.1-Acaryochloris_sp._CCMEE_5410 170
ABW30842.1-Acaryochloris_marina_MBIC11017 170
ACK69113.1-Cyanothece_sp._PCC_7424 165
EKV01940.1_Leptolyngbya_sp._PCC_7375 172

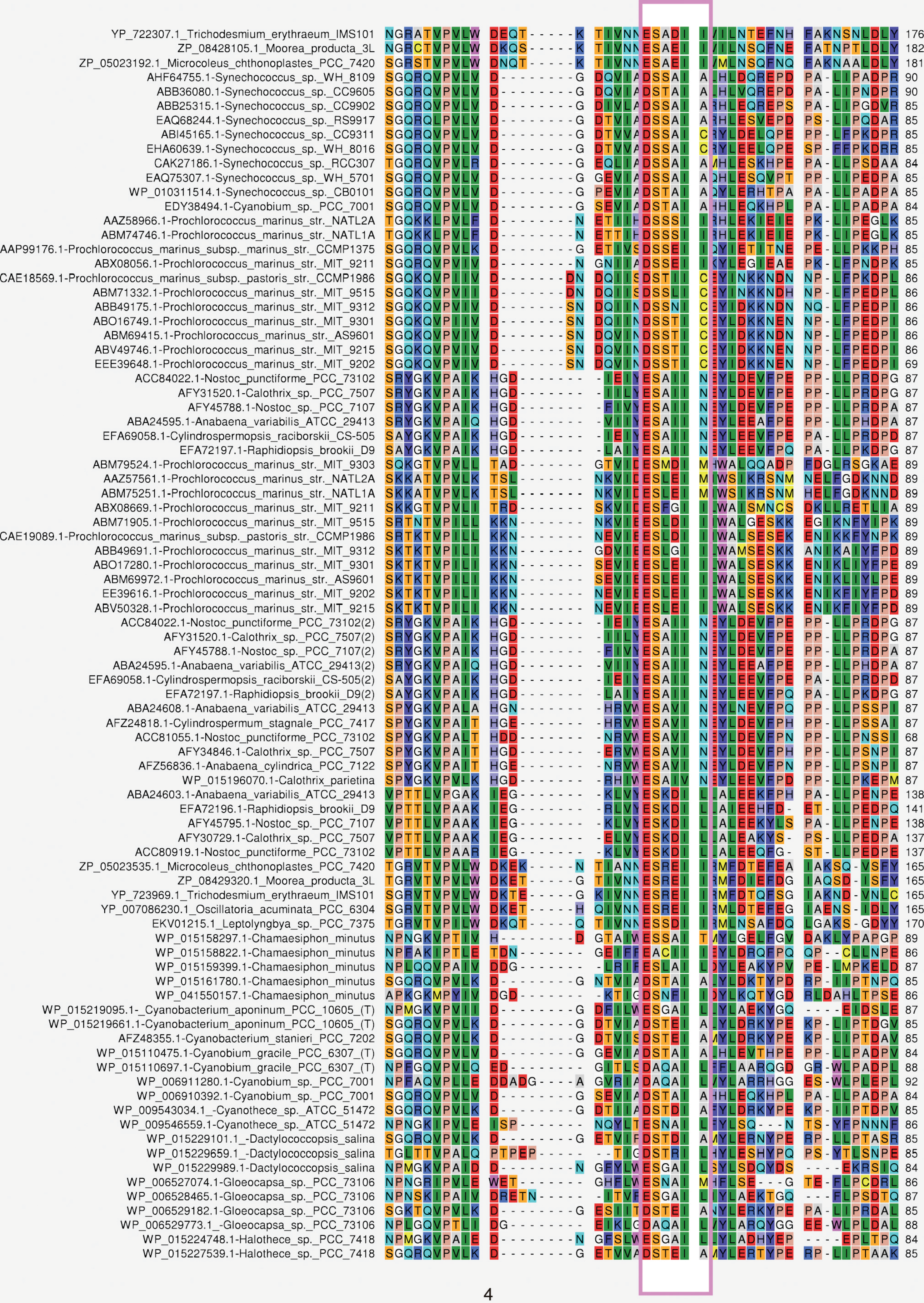
YP_722307.1_Trichodesmium_erythraeum_IMS101 176
ZP_08428105.1_Moorea_producta_3L 182
ZP_05023192.1_Microcoleus_chthonoplastes_PCC_7420 181
AHF64755.1-Synechococcus_sp._WH_8109 90
ABB36080.1-Synechococcus_sp._CC9605 90
ABB25315.1-Synechococcus_sp._CC9902 85
EAQ68244.1-Synechococcus_sp._RS9917 85
ABI45165.1-Synechococcus_sp._CC9311 85
EHA60639.1-Synechococcus_sp._WH_8016 85
CAK27186.1-Synechococcus_sp._RCC307 84
EAQ75307.1-Synechococcus_sp._WH_5701 85
WP_010311514.1-Synechococcus_sp._CB0101 85
EDY38494.1-Cyanobium_sp._PCC_7001 84
AAZ58966.1-Prochlorococcus_marinus_str._NATL2A 85
ABM74746.1-Prochlorococcus_marinus_str._NATL1A 85
AAP99176.1-Prochlorococcus_marinus_subsp._marinus_str._CCMP1375 85
ABX08056.1-Prochlorococcus_marinus_str._MIT_9211 85
CAE18569.1-Prochlorococcus_marinus_subsp._pastoris_str._CCMP1986 86
ABM71332.1-Prochlorococcus_marinus_str._MIT_9515 86
ABB49175.1-Prochlorococcus_marinus_str._MIT_9312 86
ABO16749.1-Prochlorococcus_marinus_str._MIT_9301 86
ABM69415.1-Prochlorococcus_marinus_str._AS9601 86
ABV49746.1-Prochlorococcus_marinus_str._MIT_9215 86
EEE39648.1-Prochlorococcus_marinus_str._MIT_9202 69
ACC84022.1-Nostoc_punctiforme_PCC_73102 87
AFY31520.1-Calothrix_sp._PCC_7507 87
AFY45788.1-Nostoc_sp._PCC_7107 87
ABA24595.1-Anabaena_variabilis_ATCC_29413 87
EFA69058.1-Cylindrospermopsis_raciborskii_CS-505 87
EFA72197.1-Raphidiopsis_brookii_D9 87
ABM79524.1-Prochlorococcus_marinus_str._MIT_9303 89
AAZ57561.1-Prochlorococcus_marinus_str._NATL2A 89
ABM75251.1-Prochlorococcus_marinus_str._NATL1A 89
ABX08669.1-Prochlorococcus_marinus_str._MIT_9211 89
ABM71905.1-Prochlorococcus_marinus_str._MIT_9515 89
CAE19089.1-Prochlorococcus_marinus_subsp._pastoris_str._CCMP1986 89
ABB49691.1-Prochlorococcus_marinus_str._MIT_9312 89
ABO17280.1-Prochlorococcus_marinus_str._MIT_9301 89
ABM69972.1-Prochlorococcus_marinus_str._AS9601 89
EE39616.1-Prochlorococcus_marinus_str._MIT_9202 89
ABV50328.1-Prochlorococcus_marinus_str._MIT_9215 89
ACC84022.1-Nostoc_punctiforme_PCC_73102(2) 87
AFY31520.1-Calothrix_sp._PCC_7507(2) 87
AFY45788.1-Nostoc_sp._PCC_7107(2) 87
ABA24595.1-Anabaena_variabilis_ATCC_29413(2) 87
EFA69058.1-Cylindrospermopsis_raciborskii_CS-505(2) 87
EFA72197.1-Raphidiopsis_brookii_D9(2) 87
ABA24608.1-Anabaena_variabilis_ATCC_29413 87
AFZ24818.1-Cylindrospermum_stagnale_PCC_7417 87
ACC81055.1-Nostoc_punctiforme_PCC_73102 68
AFY34846.1-Calothrix_sp._PCC_7507 87
AFZ56836.1-Anabaena_cylindrica_PCC_7122 87
WP_015196070.1-Calothrix_parietina 87
ABA24603.1-Anabaena_variabilis_ATCC_29413 138
EFA72196.1-Raphidiopsis_brookii_D9 141
AFY45795.1-Nostoc_sp._PCC_7107 138
AFY30729.1-Calothrix_sp._PCC_7507 137
ACC80919.1-Nostoc_punctiforme_PCC_73102 137
ZP_05023535.1_Microcoleus_chthonoplastes_PCC_7420 165
ZP_08429320.1_Moorea_producta_3L 165
YP_723969.1_Trichodesmium_erythraeum_IMS101 165
YP_007086230.1_Oscillatoria_acuminata_PCC_6304 165
EKV01215.1_Leptolyngbya_sp._PCC_7375 170
WP_015158297.1-Chamaesiphon_minutus 89
WP_015158822.1-Chamaesiphon_minutus 86
WP_015159399.1-Chamaesiphon_minutus 87
WP_015161780.1-Chamaesiphon_minutus 85
WP_041550157.1-Chamaesiphon_minutus 86
WP_015219095.1-_Cyanobacterium_aponinum_PCC_10605_(T) 87
WP_015219661.1-_Cyanobacterium_aponinum_PCC_10605_(T) 85
AFZ48355.1-Cyanobacterium_stanieri_PCC_7202 85
WP_015110475.1-Cyanobium_gracile_PCC_6307_(T) 84
WP_015110697.1-Cyanobium_gracile_PCC_6307_(T) 88
WP_006911280.1-Cyanobium_sp._PCC_7001 92
WP_006910392.1-Cyanobium_sp._PCC_7001 84
WP_009543034.1_-Cyanothece_sp._ATCC_51472 85
WP_009546559.1-Cyanothece_sp._ATCC_51472 86
WP_015229101.1_-Dactylococcopsis_salina 85
WP_015229659.1_-Dactylococcopsis_salina 85
WP_015229989.1-Dactylococcopsis_salina 84
WP_006527074.1-Gloeocapsa_sp._PCC_73106 86
WP_006528465.1-Gloeocapsa_sp._PCC_73106 87
WP_006529182.1-Gloeocapsa_sp._PCC_73106 85
WP_006529773.1_-Gloeocapsa_sp._PCC_73106 88
WP_015224748.1-Halothece_sp._PCC_7418 84
WP_015227539.1-Halothece_sp._PCC_7418 85

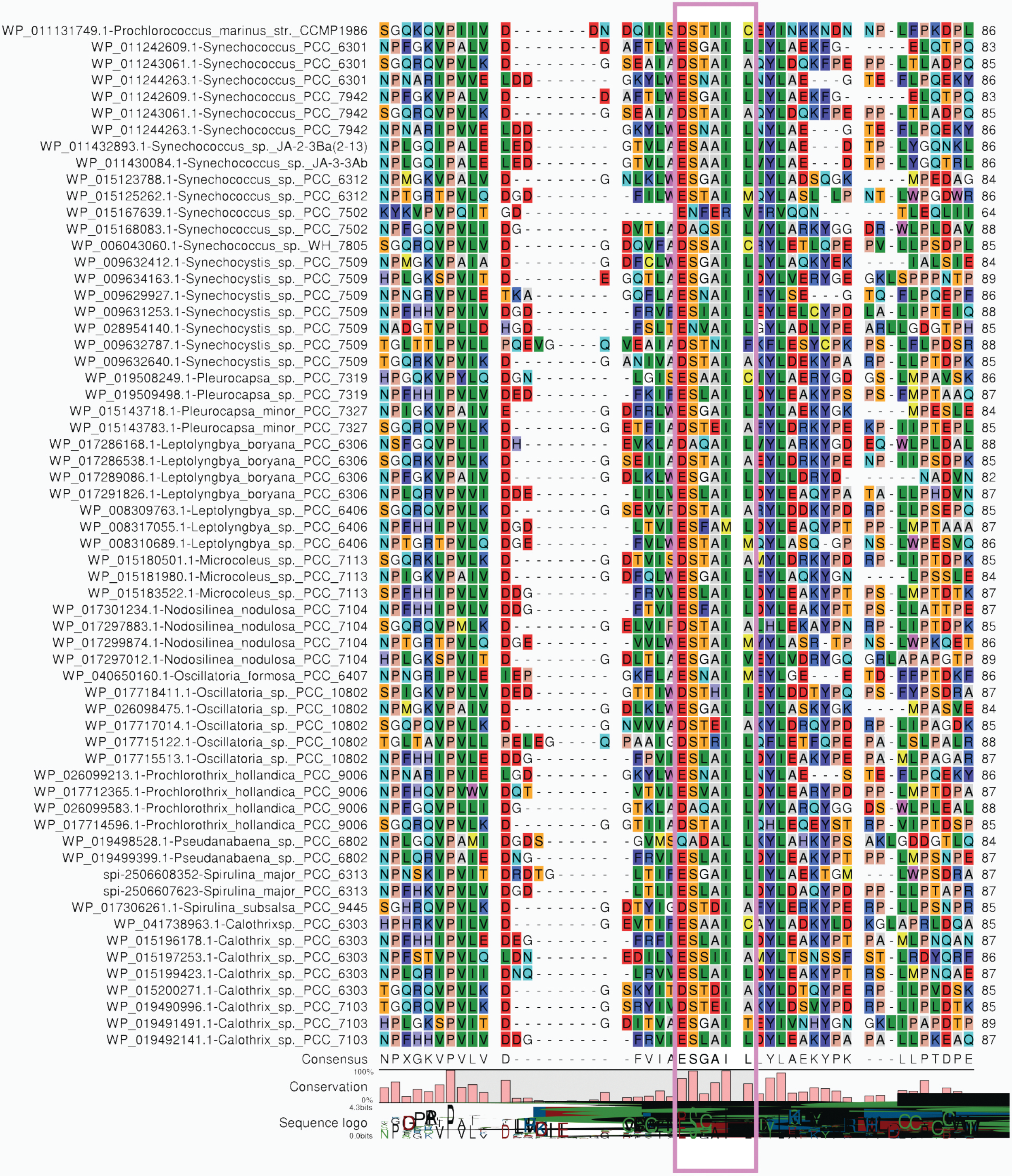

Figure S1 : Multiple sequence alignment of GST sequence found in 126 cyanobacterial sequences showing the GST motif architecture of "ES [GLNTARS][ADE]I[LAI]". Conserved domain is highlighted in pink box. Sequence alignment was performed as mentioned in section 2.2.

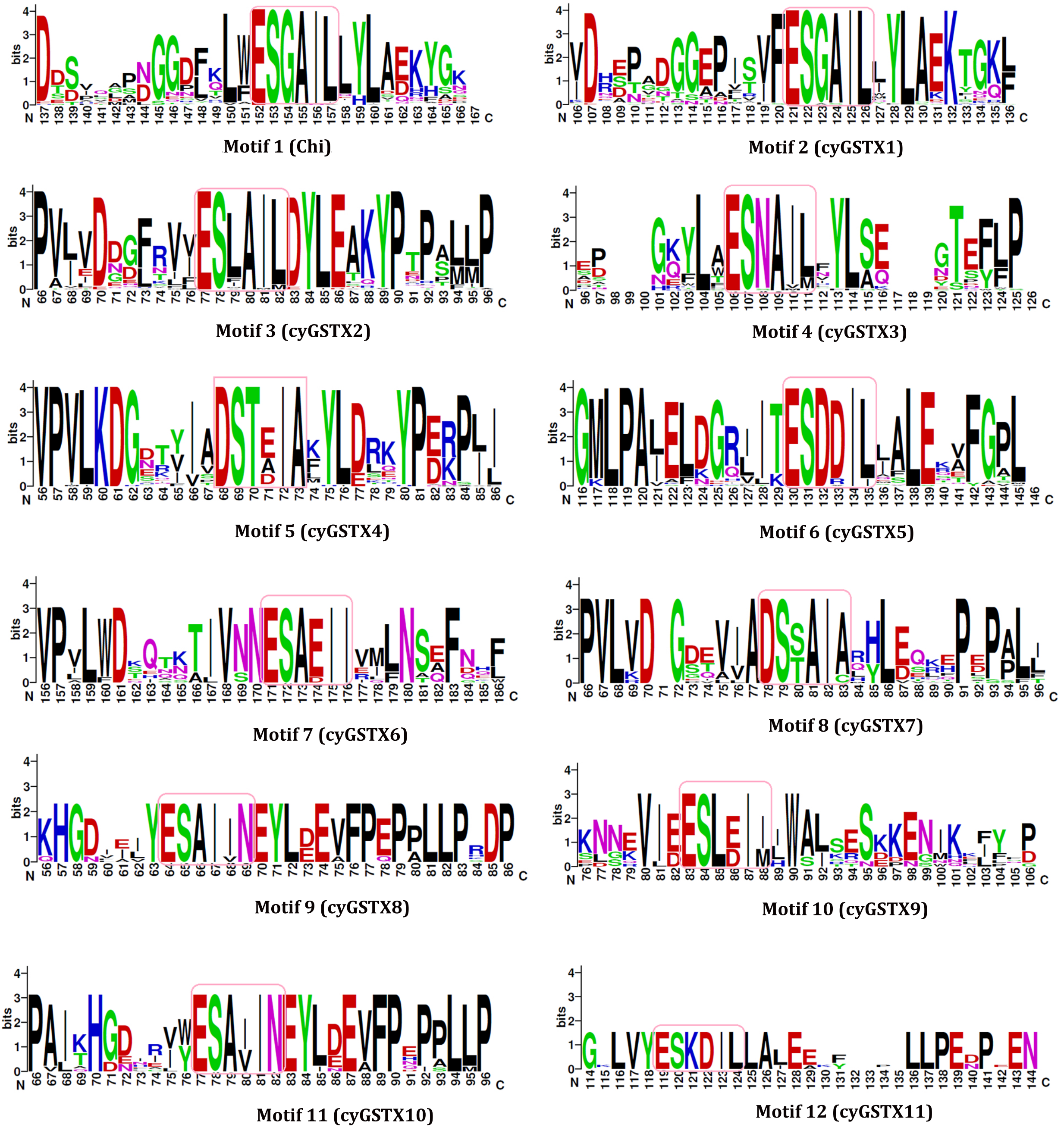

Figure S2: Highlight of the 12 GST motifs identified in retrieved GST sequences belonging to five orders of cyanobacteria. Sequence belonging to each clade was removed manually and performed sequence alignment as mentioned in section 2.2. The sequence logo was generated by subjecting the aligned file to Weblogo online server .
